# Effect of miR-17 on *Polygonum Cillinerve* polysaccharide against transmissible gastroenteritis virus

**DOI:** 10.3389/fvets.2024.1360102

**Published:** 2024-02-20

**Authors:** Xueqin Duan, Mengxin Xu, Yunying Wang, Nishang Liu, Xingchen Wang, Yingqiu Liu, Weimin Zhang, Wuren Ma, Lin Ma, Yunpeng Fan

**Affiliations:** ^1^College of Veterinary Medicine, Northwest A&F University, Yangling, China; ^2^School of Integrative Medicine, Tianjin University of Traditional Chinese Medicine, Tianjin, China

**Keywords:** *Polygonum Cillinerve* polysaccharide, transmissible gastroenteritis virus, miR-17, apoptosis, virus replication

## Abstract

Transmissible gastroenteritis virus (TGEV) could cause diarrhea, vomiting, dehydration and even death in piglets, miRNA played an important role in the interaction between virus and cell. The study aimed to investigate the impact of miR-17 on the polysaccharide of Polygonum Cillinerve (PCP) in combating TGEV. miR-17 was screened and transfection validation was performed by Real-time PCR. The function of miR-17 on PK15 cells infected with TGEV and treated with PCP was investigated by DCFH-DA loading probe, JC-1 staining and Hoechst fluorescence staining. Furthermore, the effect of miR-17 on PCP inhibiting TGEV replication and apoptosis signaling pathways during PCP against TGEV infection was measured through Real-time PCR and Western blot. The results showed that miR-17 mimic and inhibitor could be transferred into PK15 cells and the expression of miR-17 significantly increased and decreased respectively compared with miR-17 mimic and inhibitor (*P* < 0.05). A total 250 μg/mL of PCP could inhibit cells apoptosis after transfection with miR-17. PCP (250 μg/mL and 125 μg/mL) significantly inhibited the decrease in mitochondrial membrane potential induced by TGEV after transfection with miR-17 (*P* < 0.05). After transfection of miR-17 mimic, PCP at concentrations of 250 μg/mL and 125 μg/mL significantly promoted the mRNA expression of P53, cyt C and caspase 9 (*P* < 0.05). Compared with the control group, the replication of TGEV gRNA and gene N was significantly inhibited by PCP at concentrations of 250 μg/mL and 125 μg/mL after transfection of both miR-17 mimic and inhibitor (*P* < 0.05). PCP at 62.5 μg/mL significantly inhibited the replication of gene S following transfection with miR-17 inhibitor (*P* < 0.05). These results suggested that PCP could inhibit the replication of TGEV and apoptosis induced by TGEV by regulating miR-17.

## 1 Introduction

MicroRNA (miRNA) is the short RNA molecule with the size of 19–25 nucleotides, which has the ability to regulate gene expression at the post transcriptional level by inhibiting messenger RNA (mRNA) translation or promoting mRNA degradation (1). Meanwhile, miRNAs, as an important class of non-coding RNAs (2), play an important role in virus cell interactions (3). Virus infection could lead to a series of changes in cells. For example, duck pestis virus could induce cell cycle arrest at the S phase, leading to apoptosis through the activation of caspases and the increase of intracellular ROS level (4); However, drugs could improve these changes. For example, polysaccharides derived from the Antarctic green algae in Duvelia could reduce cell apoptosis, significantly inhibit the production of pro-inflammatory cytokines, and achieve the ability to inhibit H1N1 infection (5); Cephalosporin and curcumin could alleviate mitochondrial apoptosis induced by porcine circovirus type 2 by reducing ROS and MMP levels to varying degrees (6).

Transmissible gastroenteritis (TGE) is an acute, highly contagious intestinal infectious disease caused by the porcine transmissible gastroenteritis virus (TGEV). The main clinical symptoms caused by TGEV include diarrhea, vomiting, dehydration, and progressive weight loss, resulting in severe economic losses for the global pig industry (7, 8). At present, there is no effective antiviral drug available to treat this disease. Therefore, it is imperative to strengthen the drug development of TGEV. *Polygonum Cillinerve* is a commonly used Chinese medicine with functions such as hypothermia and detoxifying, promoting blood circulation, stopping bleeding and diarrhea, relieving rheumatism and strengthening the waist and knees. In clinical practice, *Polygonum Cillinerve* is widely used to treat acute stomach pain, gastroenteritis, dysentery, tonsillitis, urinary tract infections and falls, rheumatic back pain, and so on Liu et al. (9). The extract of *Polygonum Cillinerve* has also been proven to have strong antiviral activity, direct inactivation of influenza virus PR8 strain, significant inhibition of NDV proliferation in chicken embryos, and *in vitro* inhibition of HSV - I and HSV - II virus replication (10–13). In addition, *Polygonum Cillinerve* also has the effects of anti-parainfluenza type I virus, adenovirus, vesicular stomatitis virus, hepatitis B virus and so on Ma (14). *Polygonum Cillinerve* polysaccharide (PCP) is a major active component of *Polygonum Cillinerve*. Modern pharmacological studies have demonstrated that PCP exhibits significant effects as antioxidant, antibacterial, anticancer and antiviral (15, 16). Previous research has revealed that PCP was primarily composed of glucose, it was α-d-glucan and the backbone was consisted of repeating units of (1 → 4)-α-d-glucose (17). In addition, research has also shown that *Polygonum Cillinerve* polysaccharide could significantly decrease the cell apoptosis rate and the expression of ROS induced by TGEV, reduce TGEV replication, increase the expression levels of Bcl-2 and Bax mRNA, increase the expression of Bcl-2, reduce cyt C protein, and inhibit caspase 3 degradation (18).

In previous study, a target gene related to the apoptosis pathway, miR-17, was screened through the high-throughput sequencing of the expression profile changes of miRNA in PK15 cells infected with TGEV. In this study, the aim was to investigate whether treating PK15 cells with PCP and TGEV after transfection with miR-17 mimic or inhibitor would affect the changes of cell morphology, apoptosis, ROS production, and mitochondrial membrane potential. The purpose is to further explore the mechanism of PCP on inhibiting TGEV and provide the theoretical basis for the development of antiviral drugs.

## 2 Materials and methods

### 2.1 Drugs, cells, and viruses

*Polygonum Cillinerve* was collected from Taibai Mountain and preserved in the Veterinary Laboratory of Northwest A&F University. It was identified by Professor Song Xiaoping from the College of Veterinary Medicine of Northwest A&F University as the root tuber of *Polygonum Cillinerve* (Nakai) Ohwl, a plant of the polygonaceae family. *Polygonum Cillinerve* polysaccharide were prepared in the laboratory and their structures have been identified in previous study (17), TGEV was donated by Dr. Wang Xuefei from Henan Institute of Animal Husbandry Economics; PK15 cells (collection number: GDC0061) were purchased from the China Typical Culture Collection Center of Wuhan University.

### 2.2 Main reagents

DMEM high sugar medium and 3- (4,5-dimethylthiazole-2)−2,5- diphenyltetrazolium bromide (MTT), Beijing Solaybao Technology Co., Ltd; Fetal bovine serum (FBS) was purchased from Sigma Company; miR-17 mimic, miR-17 inhibitor, mimic negative control (mimic NC), inhibitor negative control (inhibitor NC) were synthesized by Guangzhou Ruibo Biotechnology Co., Ltd; Lipofectamine 2000 transfection reagent (Catalog Number: 11668019), produced by Thermo Fisher Scientific; TRIzol, 5 × Integrated RT MasterMix kit, 2 × Fast qPCR Master Mixture (Green) fluorescence quantification kit, RNA extraction kit, reverse transcription kit, products of Beijing Dining Biotechnology Co., Ltd; The primers for TGEV gRNA, TGEV N gene, miR-17, and U6 (internal reference) were designed and synthesized by Bioengineering (Shanghai) Co., Ltd; Mitochondrial membrane potential (JC-1) detection kit, reactive oxygen species detection kit, and Hoechst 33258 staining solution, produced by Beijing Solaybao Technology Co., Ltd; P53, cyt C, caspase 9, β-Actin (internal reference) primer, synthesized by Shenggong Biotechnology (Shanghai) Co., Ltd; P53 first antibody, cyt C first antibody, caspase 9 first antibody, β-Actin first antibody, sheep anti rabbit IgG, and sheep anti mouse IgG are products of CST Company in the United States.

### 2.3 Experimental methods

#### 2.3.1 Resuscitation and culture of PK15 cells

PK15 cells were removed from the liquid nitrogen tank and melt in the 37°C thermostat water bath. After completed melt, the cells were added with 3 mL of complete culture medium containing 10% fetal bovine serum and mixed completely. Next, the cells were centrifuged at 1000 r/min for 5 min, discarded the culture medium, added with the complete culture medium, and resuspended and mixed completely. These cells were transferred to the cell culture dish and cultured at 37°C and 5% CO_2_ (MCO-18AC, Puwa Corporation, Japan).

#### 2.3.2 The validation of differential miR-17 by RT-qPCR

PK15 cells were adjusted the concentration to 1.6 × 10^5^ cells/mL, inoculated into the cell plate, then cultured at 37°C and 5% CO_2_ until the cell abundance was around 80%, and then TGEV and PCP were added into the plate, the cells were incubated at 37°C for 1.5 h, then the cells were washed 3 times with PBS, and replaced with fresh maintenance medium. Meanwhile, the virus control group was set. The cells were collected to extract RNA according to TRIzol extraction kit instructions after further cultured for 48 h, and the extracted total RNA was synthesized cDNA in the reaction system and reaction procedure of the 5 × Integrated RT Master Mix Reverse Transcription Kit, and miR-17 reverse transcription primer 5 '- GTCGTTCACAGTG CAGGGTCGAGGTATCGACTACAA-3' (PCR amplification instrument, TC-XP type, Hangzhou Bori Technology Co., Ltd., China) was added; cDNA was used as the template, followed 2 × Fast Real time PCR Master Mixture (Green) kit reaction system and the procedure was used for PCR amplification (Fluorescence quantitative gene amplification instrument, CFX Connect, Bole Company, USA). The primers were shown in Table 1. The results of Real-time PCR were analyzed by 2^−Δ*ΔCt*^ method.

**Table 1:** Primer sequences.

**Gene name**	**Sequences**
miR-17	F: GCGACTGCAGTGAAGGCAC
	R: AGTGCAGGGTCCGAGGTATT
U6	F: CTCGCTTCGGCAGCACA
	R: AACGCTTCACGAATTTGCGT
gRNA	F: ACTGACCAGGAGAGGCAAACTATTG
	R: TGCCCTTAAGCTGTTCACCGTATG
TGEV N	F:GGTAGTCGTGGTGCTAATAATGAA
	R:TTGGATTGTTGCCTGCCTCTA
TGEV S	F:CTAGCACCATGTAAATAAGCAACAACCTC
	R:GAAGGGACAAAGGACGACAG
β-actin	F: GGACTTCGAGCAGGAGATGG
	R: AGGAAGGAGGGCTGGAAGAG

#### 2.3.3 The expression of miR-17 after transfection with mimic or inhibitor by Real-time PCR

PK15 cells were lay on the 12-well plates, and changed basic medium and starved for 30 min when the cells grew to 30%−40%. Following the steps in the miRNA product manual and Lipofectamine 2000 transfection reagent manual, miR-17 mimic and its corresponding NC were transfected separately at the concentration of 50 nM; The miR-17 inhibitor and its corresponding NC were transfected separately at the concentration of 100 nM. After 4 h of transfection, the cells were changed the complete medium, continued to culture for 24 h, then washed with PBS 3 times, added with 0.5 mL of TRIpure Reagent to extract RNA. The extracted total RNA was subjected to reverse transcription and PCR amplification according to the method in 2.3.2. The results of Real-time PCR were analyzed by using 2^−Δ*ΔCt*^ method.

#### 2.3.4 Cell transfection and drug treatment

PK15 cells were placed in good condition on cell plates, and changed the basic culture medium and starved for 30 min when the cells grew to 30%−40%. Following the steps in the miRNA product manual and Lipofectamine 2000 transfection reagent manual, miR-17 mimic and its corresponding NC were transfected separately at the concentration of 50 nM. The miR-17 inhibitor and its corresponding NC were transfected separately at the concentration of 100 nM. After 4 h of transfection, the cells were changed the culture medium completely and continued to cultivate for 24 h. Then, TGEV and different concentrations of PCP (62.5 μg/mL, 125 μg/mL, 250 μg/mL) were added to the cells transfected of miR-17 mimic or inhibitor, and the cells of NC and mimic (or inhibitory) were transfected with the corresponding volume of TGEV, cultivated for 1.5 h, washed with PBS for 3 times, replaced the maintenance medium and continued to cultivate for 24 h before proceeding with subsequent operations.

#### 2.3.5 Observation of cell morphology under inverted microscope

PK15 cells were treated according to the method in 2.3.4. After PK15 cells were cultured for 24 h, the cell morphology was observed by using microscope (Inverted microscope CX23, OLYMPUS, Japan).

#### 2.3.6 Fluorescence microscopy observation of ROS production in cells

PK15 cells were treated according to the method in 2.3.4. Then PK15 cells were washed twice with serum-free DMEM, and then added with DCFH-DA loaded probes diluted with 1:1000 serum-free culture medium, and incubated in 37°C for 20 min. Next, the PK15 cells were washed for 3 times with serum-free cell culture medium to fully remove DCFH-DA that has not entered the cells. Finally, the cells were taken randomly from different fields of view to take photographs and record (Fluorescent Inverted Microscope ICX41, Shunyu Optical Technology Co., Ltd., China).

#### 2.3.7 Fluorescence staining method for observing mitochondrial membrane potential

PK15 cells were treated following the procedure outlined in 2.3.4. Then 1 mL of complete culture medium and 1 mL of JC-1 staining working solution were added into cells after the culture medium was gently sucked out and washed with PBS once per well. Following the incubation, the supernatant was removed and the cells were washed twice with JC-1 staining buffer in ice bath, added with 2 mL of complete culture medium. Finally, the cells were observed and photographed by the inverted fluorescence microscope, and then the fluorescence intensity was analyzed.

#### 2.3.8 Hoechst 33258 staining for cell apoptosis detection

PK15 cells were treated according to the method in 2.3.4. Then the culture medium from each well was gently sucked out, and the cells were carefully washed with PBS 3 times, added with 0.5 mL of fixative to fix at room temperature for 15 min. Next, fixative was removed and the cells were washed with PBS for three times. After sucking out all the liquid, 0.5 mL of Hoechst 33258 staining solution was added into cells for equilibrating at room temperature 30 min and incubated for 5 min before shaking. Following the removal of removing the staining solution, the cells were cleaned twice with PBS for 3 min each time and sucked out all the liquid. Finally, the anti-fluorescence quenching sealing solution was added one drop of per well, and the cells were observed and photographed under the inverted fluorescence microscope (UV).

#### 2.3.9 Real-time PCR detection of expression levels of P53, cyt C, and caspase 9 at mRNA level

According to the methods of 2.3.2, the RNA was extracted by TRIzol extraction kit, and the extracted total RNA was synthesized cDNA in the reaction system and procedure of the 5 × Integrated RT Master Mix Reverse Transcription Kit. PCR amplification was performed according to the reaction system and procedure of the 2 × Fast Real time PCR Master Mixture (Green) kit using cDNA as templates. Primers were shown in Table 2. The results of Real-time PCR were analyzed by using 2^−Δ*ΔCt*^ method.

**Table 2:** List of Real-time PCR primers.

**The purpose gene**	**Primer sequences**	**Size**
cyt C	F: CTCTTACACAGATGCCAACAA	139
	R: TTCCCTTTCTCCCTTCTTCT	
caspase 9	F: GGACATTGGTTCTGGAGGATT	116
	R: TGTTGATGATGAGGCAGTGG	
P53	F: CGAACTGGCTGGATGAAAAT	124
	R: GAAGGGACAAAGGACGACAG	
β-actin	F: GGACTTCGAGCAGGAGATGG	138
	R: AGGAAGGAGGGCTGGAAGAG	

#### 2.3.10 Detection of the expression of key signal molecules in the apoptosis pathway by western blot

PK15 cells were treated according to the method in 2.3.4. After cultured for 24 h, the cells were centrifuged after trypsin digestion, and the supernatant was removed. And then, the cells were added with 100 μL of lysis solution, splited on ice for 1 h, centrifuged at 12000 r/min for 15 min, and then the supernatant was taken away. The concentration of the protein in the supernatant was measured by using BCA method and adjusted. Next, 5 × protein loading buffer (including DTT) instruction manual was used for protein inactivation treatment, and it was stored at −20°C after inactivation. The denatured protein was added into the electrophoresis device, and the voltage was set to 80 V initially, then the voltage was changed to 120 V for 80 min when the sample ran between the concentrated gel and the separation gel (about 20 min). After electrophoresis, the gel was removed, the film was transferred. The installed film transfer clamp was placed in the electrophoresis tank. An appropriate amount of pre-cooled membrane transfer fluid was added, and the membrane was transferred under a constant pressure of 100 V for 66 min. After the membrane transfer was completed, the PVDF membrane was removed, rinsed in TBST solution, and then sealed in 5% skimmed milk powder prepared with TBST solution. It was shaken and sealed for 1 h at 37°C. The PVDF membrane was washed 4 times with TBST solution for 15 min each time, and then placed in primary antibody incubation solution diluted with TBST at the appropriate concentration and incubated overnight at 4°C. After washing the membrane of the first antibody for 4 times, each time for 15 min, and it was placed in the second antibody incubation solution diluted with TBST solution, then incubated at room temperature for 1 h. The membrane was incubated with the specific secondary antibody for 1 h after washing 4 times with TBST. Finally, the membrane was washed 4 times with TBST, the signal was observed with ECL (Chemiluminescence gel imager, JS-M6, Shanghai Peiqing Technology Co., Ltd., China), and the membrane was exposed on the X-ray film.

#### 2.3.11 TGEV genome and subgenome expression levels by Real-time PCR

PK15 cells were treated according to the method in 2.3.4. The RNA was extracted by TRIzol extraction kit, and the total RNA extracted was subjected to reverse transcription and PCR amplification according to the method in 2.3.2. The primers were shown in Table 1. The results of Real-time PCR were analyzed by using 2^−Δ*ΔCt*^ method.

#### 2.3.12 Data analysis

The data was analyzed using statistical software IBM SPSS Statistics 21.0. The data was expressed as mean ± standard deviation (Mean ± SD), and the One-way ANOVA was used for inter group comparison. *P* < 0.05 was statistically significant, ^*^P < 0.05, ^**^ *P* < 0.01, ^****^P < 0.001. The data and analysis results were plotted as a column chart using GraphPad Prism 7.00.

## 3 Results

### 3.1 The validation results of differentially expressed miR-17

**Figure 1:**
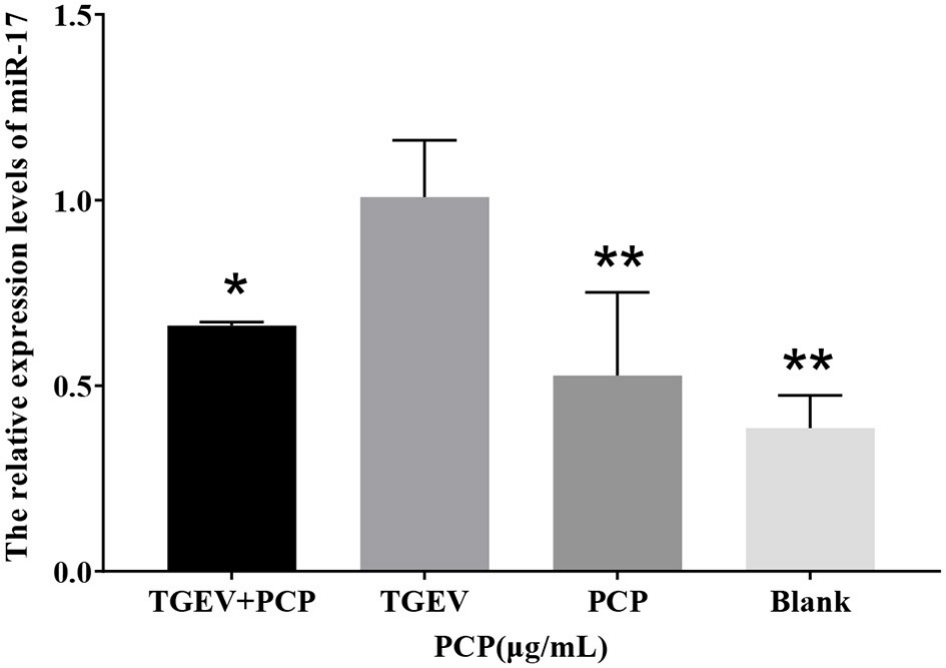
The relative expression of differential miR-17. *P* < 0.05 was statistically significant, ^*^P < 0.05, ^**^P < 0.01.

In the sequencing results, the differentially expressed miR-17 was significantly lower in the experimental group than that in the control group. The results of Real-time PCR were shown in Figure 1, and the relative expression of miR-17 in the experimental group was significantly lower than that in the TGEV control group (*P* < 0.05), consisting with the sequencing results. And the miR-17 levels in the blank control group and the PCP group were significantly lower than those in the TGEV group (*P* < 0.01).

### 3.2 Expression of miR-17 after transfection with mimic or inhibitor

**Figure 2:**
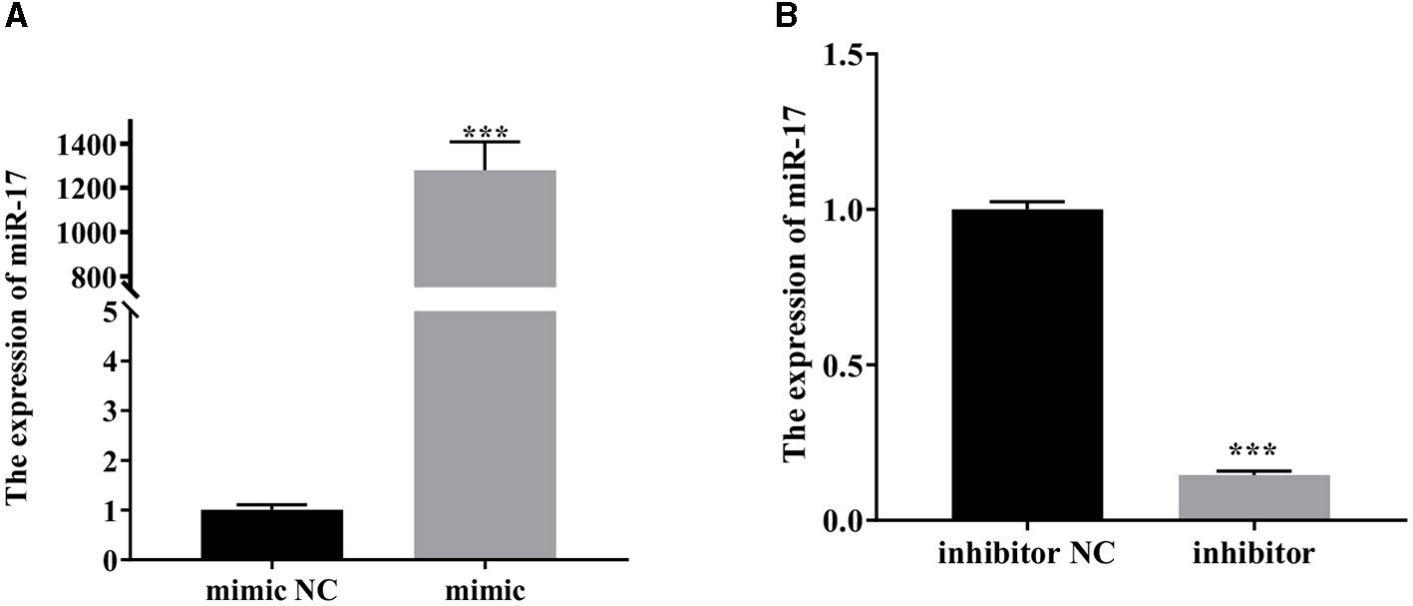
The relative expression of miR-17. The PK15 cells were treated with miR-17 mimic, mimic NC, inhibitor and inhibitor NC, respectively, and without using PCP and TGEV. **(A)** The expression level of miR-17 in PK15 cells transfected with miR-17 mimic (*n* = 3); **(B)** The expression level of miR-17 in PK15 cells transfected with miR-17 inhibitor The PK15 cells were treated with miR-17 mimic, mimic NC, inhibitor and inhibitor NC, respectively, and without using PCP and TGEV (*n* = 3). *P* < 0.05 was statistically significant, ^***^P < 0.001.

As depicted in Figure 2, the expression of miR-17 was significantly increased after transfection of miR-17 mimic compared with the mimic NC group (*P* < 0.001) (Figure 2A). Compared with the inhibitor NC group, the expression of miR-17 was significantly reduced after transfection with miR-17 inhibitor (*P* < 0.001) (Figure 2B). All groups were not treated with PCP and TGEV.

### 3.3 Changes in cell morphology after transfection with miR-17 mimic or miR-17 inhibitor

**Figure 3:**
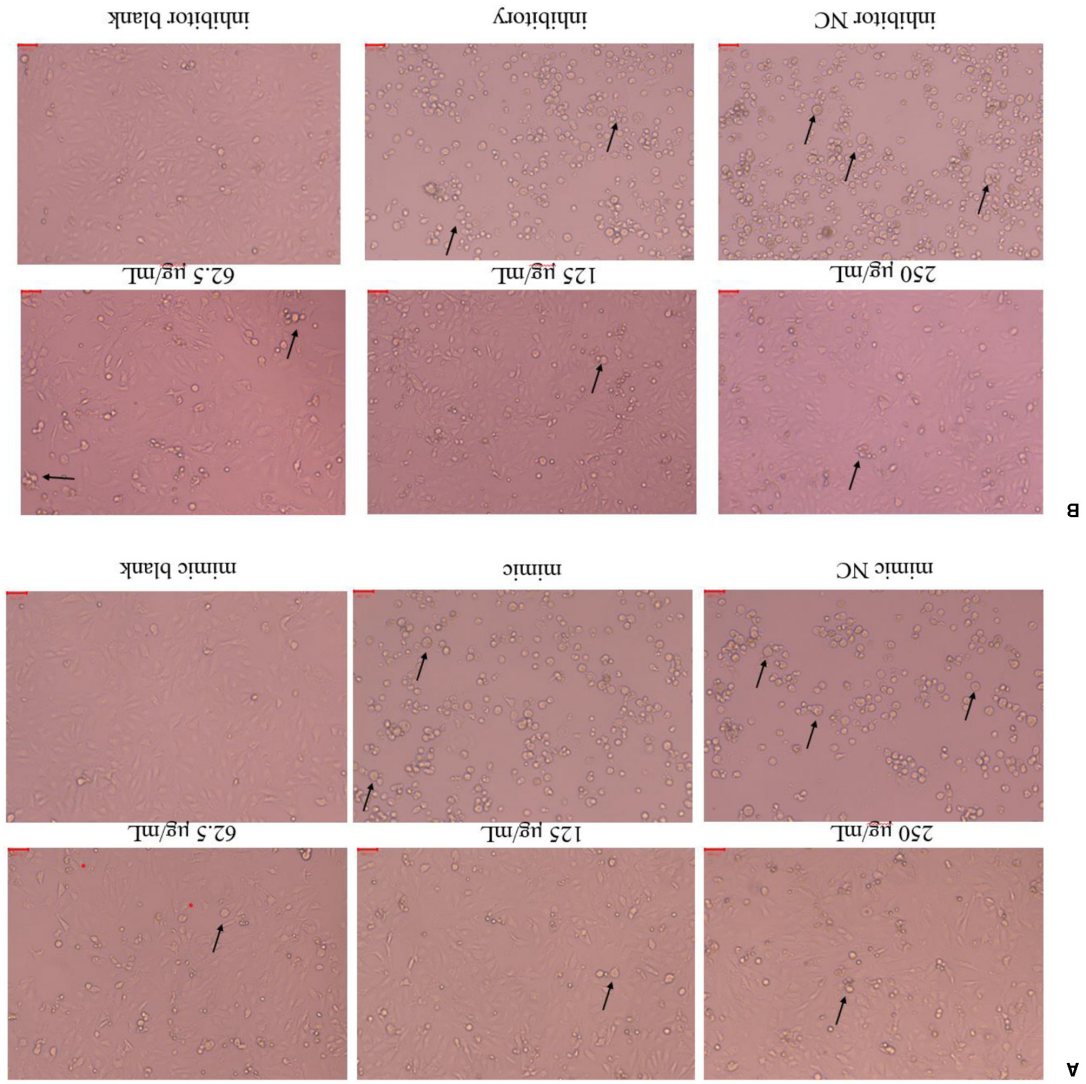
Cell morphology ( × 200). The arrow referred to the PK15 cells that have swollen, ruptured and shed into clusters. **(A)** Cell morphology of PCP groups (250, 125, 62.5 μg/mL) transfected with miR-17 mimic, mimic group, mimic NC group and mimic blank group ( × 200); **(B)** Cell morphology of PCP groups (250, 125, 62.5 μg/mL) transfected with miR-17 inhibitor, inhibitor group, inhibitor NC group and inhibitor blank group ( × 200). The scale was 20 μm.

The cell morphology is shown in Figure 3. The PK15 cells in the blank group were uniformly sized, irregularly polygonal and tightly connected together, which had very few floating cells; The PK15 cells in the 250 μg/mL and 125 μg/mL PCP groups had the homogeneous cell morphology and could form a monolayer of cells with a small number of cells floating; The PK15 cells in the PCP group (62.5 μg/mL) grew slowly and did not form a continuous monolayer of adherent cells, but there were a small number of cell clusters; The PK15 cells in the NC and control groups were circular, fragmented, and detached, with a very small number of adherent cells observed. However, the floating cells in the mimic NC group were fewer than those in the mimic group (Figure 3A), on the contrary, the floating cells in the inhibitor NC group were more than those in the inhibitor group (Figure 3B). Therefore, it could be seen that miR-17 had an impact on the cell morphology after TGEV infection; At the same time, after simultaneous transfection of miR-17, the drug groups had a good inhibitory effect on TGEV.

### 3.4 Effects on ROS after transfection with miR-17 mimic or miR-17 inhibitor

**Figure 4:**
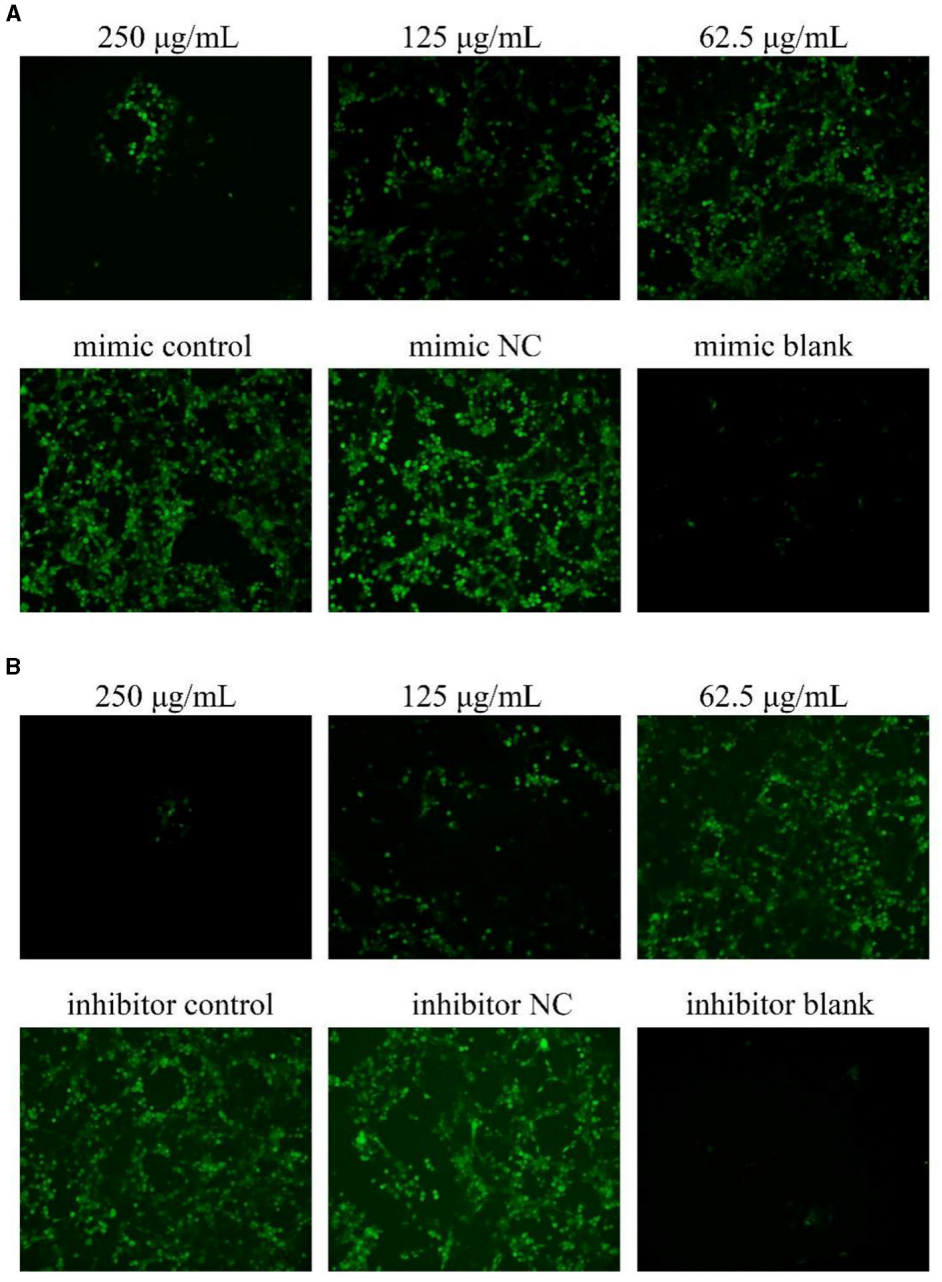
ROS production in PK15 cells. The level of ROS production in PK15 cells was detected by DCFH-DA. The green part represented ROS levels. PCP, mimic, mimic NC, inhibitor and inhibitor NC groups were treated with TGEV while blank group was untreated. **(A)** ROS level of PCP groups (250, 125, 62.5 μg/mL) transfected with miR-17 mimic, mimic group, mimic NC group and mimic blank group ( × 200); **(B)** ROS level of PCP groups (250, 125, 62.5 μg/mL) transfected with miR-17 inhibitor, inhibitor group, inhibitor NC group and inhibitor blank group ( × 200).

As shown in Figure 4, after transfection with miR-17 mimic and inhibitor, the green fluorescence of the NC group was higher than that of the control group, and the green fluorescence of the PCP groups (250 μg/mL−62.5 μg/mL) were lower than that of the control group, and the lower the drug concentration, the more fluorescence (Figures 4A, B). It revealed that transfection of miR-17 inhibited the production of ROS caused by TGEV; Meanwhile, PCP inhibited the production of ROS induced by TGEV after transfection of miR-17.

### 3.5 Effect of transfection of miR-17 mimic or inhibitor on mitochondrial membrane potential changes

**Figure 5:**
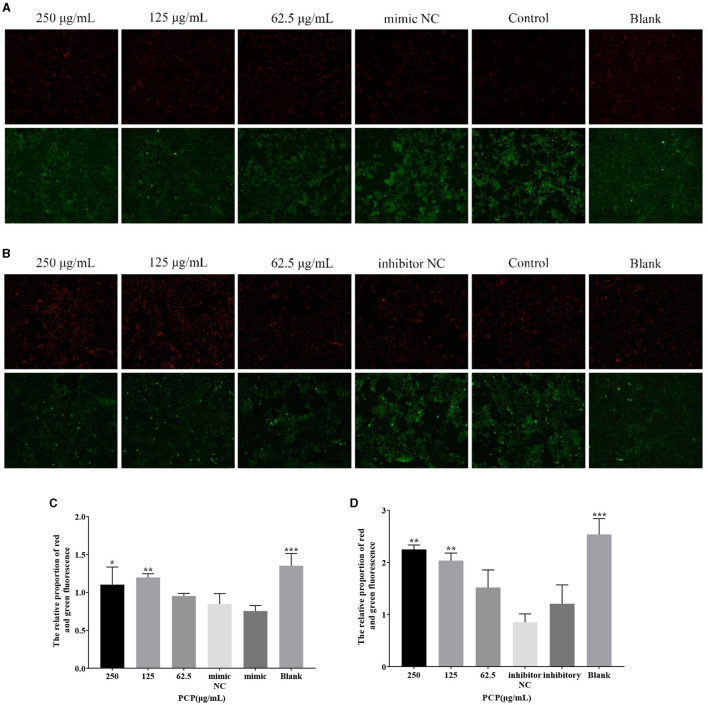
Mitochondrial membrane potential. Mitochondrial membrane potential of PK15 cells was detected by JC-1. The red part represented normal mitochondria, and the green part represented mitochondria with decreased membrane potential. The PCP, mimic, mimic NC, inhibitor and inhibitor NC groups were treated with TGEV while blank group was untreated. **(A)** Mitochondrial membrane potential of PCP groups (250, 125, 62.5 μg/mL) transfected with miR-17 mimic, mimic NC group, mimic group and blank group ( × 200); **(B)** Mitochondrial membrane potential of PCP groups (250, 125, 62.5 μg/mL) transfected with miR-17 inhibitor, inhibitor group, inhibitor NC group and inhibitor blank group ( × 200); **(C)** The relative ratio of red and green fluorescence of PCP groups transfected with miR-17 mimic; **(D)** The relative ratio of red and green fluorescence of PCP groups transfected with miR-17 inhibitor. *P* < 0.05 was statistically significant, *P < 0.05, **P < 0.01, ***P < 0.001.

The results were shown in Figure 5. From Figure 5A, it could be seen that after transfection of miR-17 mimic, the red fluorescence of the 250 μg/mL and the blank groups were the highest compared to the other groups, while the green fluorescence of the mimic NC and mimic groups were also the highest compared to the other groups. After analysis and calculation of the red and green fluorescence ratio of each group in Figure 5A, there was no difference between the mimic NC group and the mimic group. The red and green fluorescence ratio of the blank group was significantly higher than that of the mimic group (*P* < 0.001), and at the same time, the red and green fluorescence ratio of the 250 μg/mL group and 125 μg/mL group was significantly higher than that of the mimic group (*P* < 0.05) (Figure 5C). After transfection of miR-17 inhibitor, the red fluorescence of the 250 μg/mL group and 125 μg/mL group was the highest compared to the other groups, while the green fluorescence of the inhibitor NC and inhibitory groups was also the highest compared to the other groups. According to analysis and calculation of the red and green fluorescence ratio of each group of cells in Figure 5B, there was no difference between the inhibitor NC group and the inhibitory group, the red and green fluorescence ratio of the 250 μg/mL group, 125 μg/mL group and the blank group was significantly higher than that of the inhibitory group (*P* < 0.05) (Figure 5D). The transfection of miR-17 mimic and inhibitor did not affect the changes in mitochondrial membrane potential caused by TGEV; Meanwhile, PCP inhibited the phenomenon of decreased mitochondrial membrane potential in PK15 cells infected with TGEV after transfection with miR-17 mimic or inhibitor.

### 3.6 Effects of miR-17 mimic or inhibitor transfection on cell apoptosis

**Figure 6:**
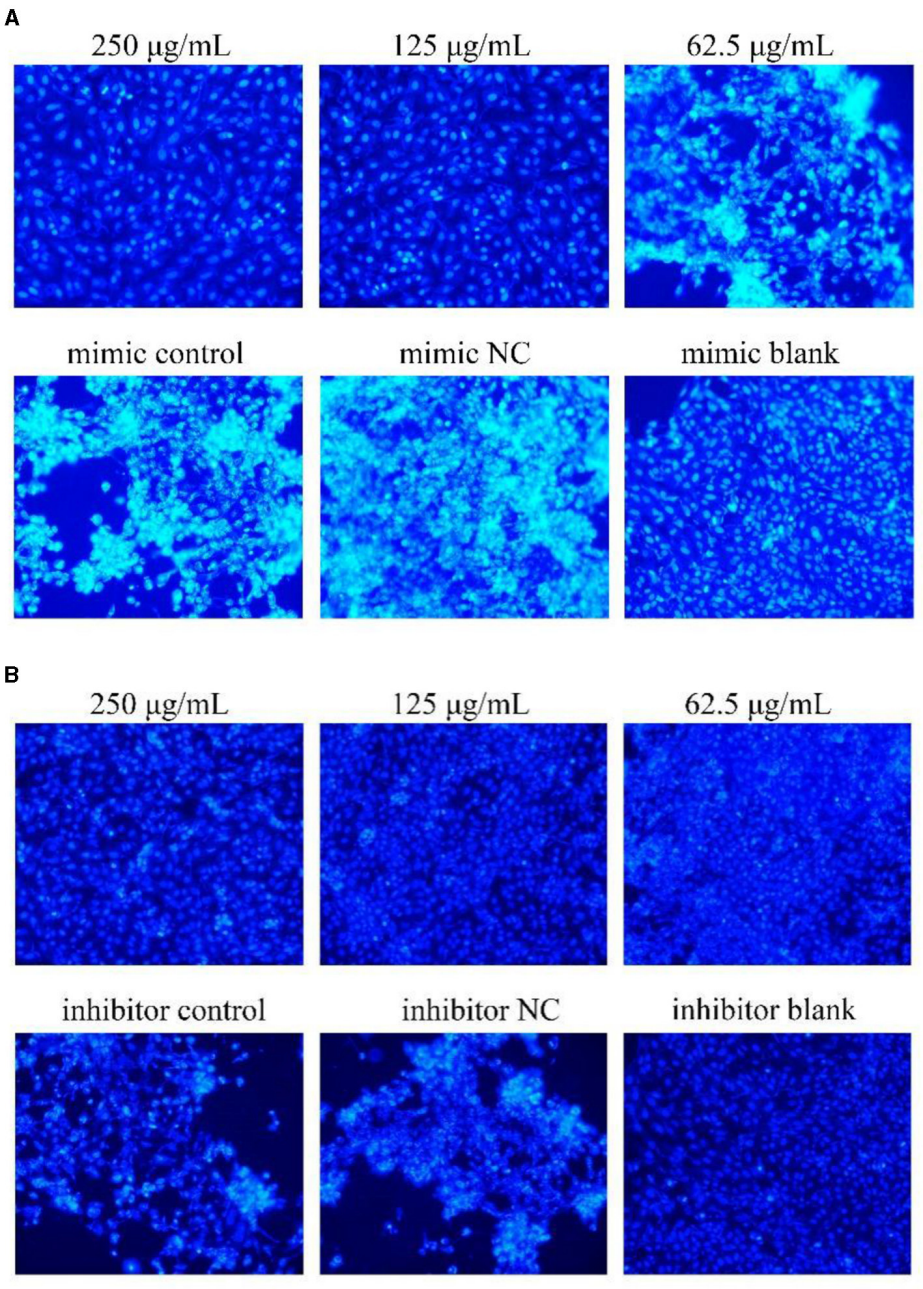
Hoechst 33258 detected the effect on cell apoptosis ( × 200). The white part represented apoptotic cells. The PCP, mimic, mimic NC, inhibitor and inhibitor NC groups were treated with TGEV while blank group was untreated. **(A)** Apoptosis level of PCP groups (250, 125, 62.5 μg/mL) transfected with miR-17 mimic, mimic group, mimic NC group and mimic blank group ( × 200); **(B)** Apoptosis level of PCP groups (250, 125, 62.5 μg/mL) transfected with miR-17 inhibitor, inhibitor group, inhibitor NC group and inhibitor blank group ( × 200).

The results were shown in Figure 6. After transfection of miR-17 mimic, the nuclei of the miR-17 mimic NC group were whiter than those of the mimic group, and the cell states of the 250, 125 μg/mL and the blank groups were observed to be optimal without significant nuclear whitening, indicating minimal cell apoptosis; The phenomenon of cell apoptosis was significant in the 62.5 μg/mL group, and the phenomenon of nuclear whitening was weaker than that in the mimic group (Figure 6A). After transfection of miR-17 inhibitor, inhibitor NC had more whitish nuclei than inhibitory group. Compared with the inhibitory group, the 250 μg/mL−62.5 μg/mL groups showed no significant nuclear whitening and fewer apoptotic cells (Figure 6B).

### 3.7 Effect of transfection with miR-17 mimic or inhibitor on mRNA expression of key signaling molecules

**Figure 7:**
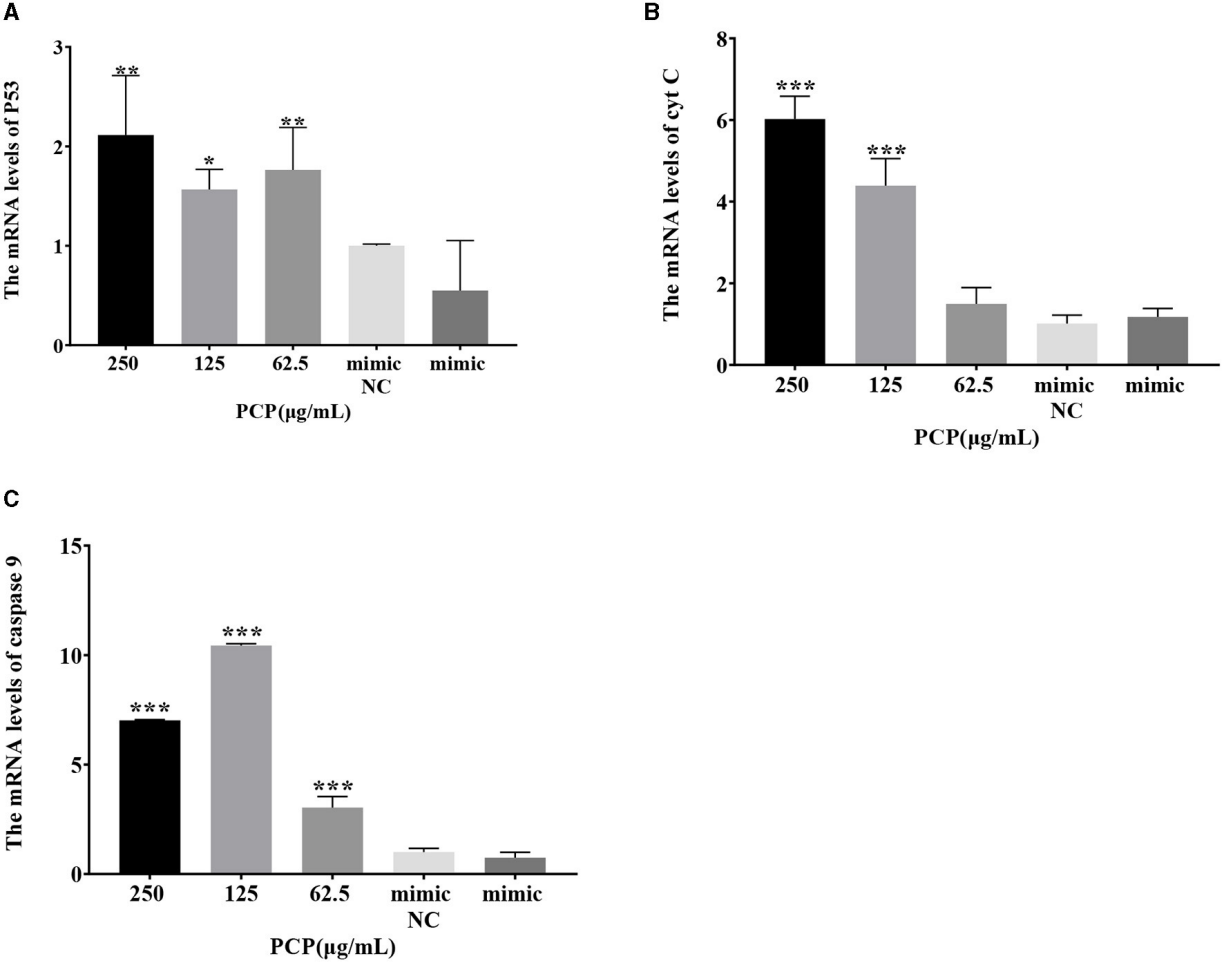
Effect of expression of key signaling molecule after transfection of miR-17 mimic. The mRNA expression levels of P53, cyt C and caspase-9 were measured by RT-qPCR. The PCP, mimic and mimic NC groups were treated with TGEV. **(A)** The mRNA expression level of P53 (*n* = 3); **(B)** The mRNA expression level of cyt C (*n* = 3); **(C)** The mRNA expression level of caspase-9 (*n* = 3). *P* < 0.05 was statistically significant to the mimic group, *P < 0.05, **P < 0.01, ***P < 0.001.

**Figure 8:**
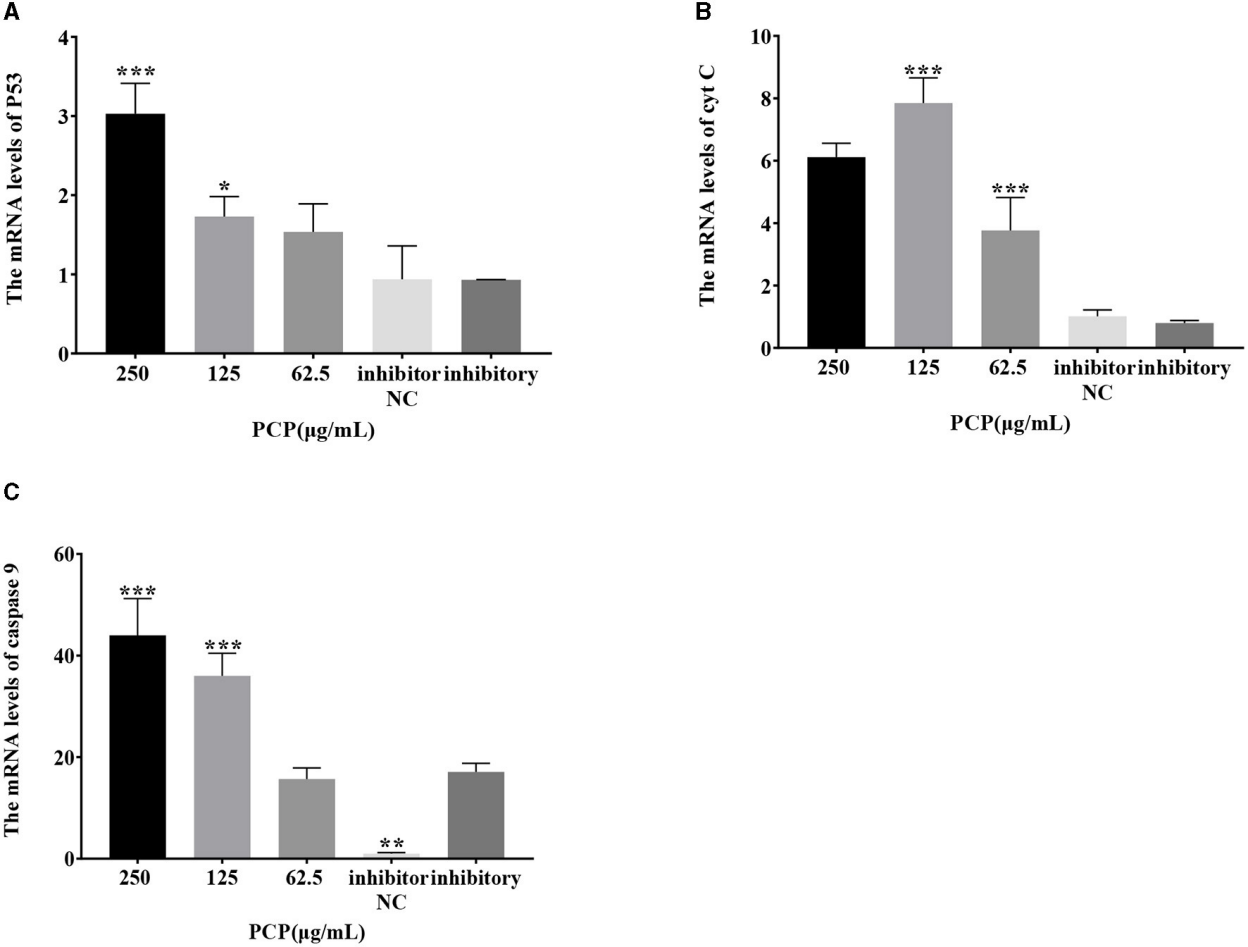
Effect of expression of key signaling molecule after transfection of miR-17 inhibitor. The mRNA expression levels of P53, cyt C and caspase-9 were measured by RT-PCR. The PCP, inhibitory and inhibitor NC groups were treated with TGEV. **(A)** The mRNA expression level of P53 (*n* = 3); **(B)** The mRNA expression level of cyt C (*n* = 3); **(C)** The mRNA expression level of caspase-9 (*n* = 3). *P* < 0.05 was statistically significant to the inhibitory group, *P < 0.05, **P < 0.01, ***P < 0.001.

The results were shown in Figure 7. After transfection of miR-17 mimic, no difference was observed in the mRNA expression of P53, cyt C, and caspase 9 between the mimic group and the mimic NC group; The PCP groups(250 and 62.5 μg/mL) significantly promoted the mRNA expression of P53 (*P* < 0.01); The PCP group of 125 μg/mL significantly promoted the mRNA expression of P53 (*P* < 0.05) (Figure 7A); The PCP groups of 250 and 125 μg/mL significantly promoted mRNA expression of cyt C (*P* < 0.001) (Figure 7B); The PCP groups of 250 μg/mL−62.5 μg/mL significantly promoted the expression of mRNA of caspase 9 (*P* < 0.001) (Figure 7C). After transfection of miR-17 inhibitor, the mRNA expression of caspase 9 in the inhibitor NC group was significantly lower than that in the inhibitory group (*P* < 0.01) (Figure 8C); The PCP group of 250 μg/mL significantly promoted the expression of P53 mRNA (*P* < 0.001) (Figure 8A); The PCP group of 125 μg/mL significantly promoted the mRNA expression of P53 (*P* < 0.05) (Figure 8A); The PCP groups of 125 and 62.5 μg/mL significantly promoted the mRNA expression of cyt C (*P* < 0.001) (Figure 8B); The PCP groups of 250 and 125 μg/mL significantly promoted the mRNA expression of caspase 9 (*P* < 0.001) (Figure 8C).

### 3.8 Effect of transfection with miR-17 mimic or miR-17 inhibitor on the expression of key signaling molecule proteins

**Figure 9:**
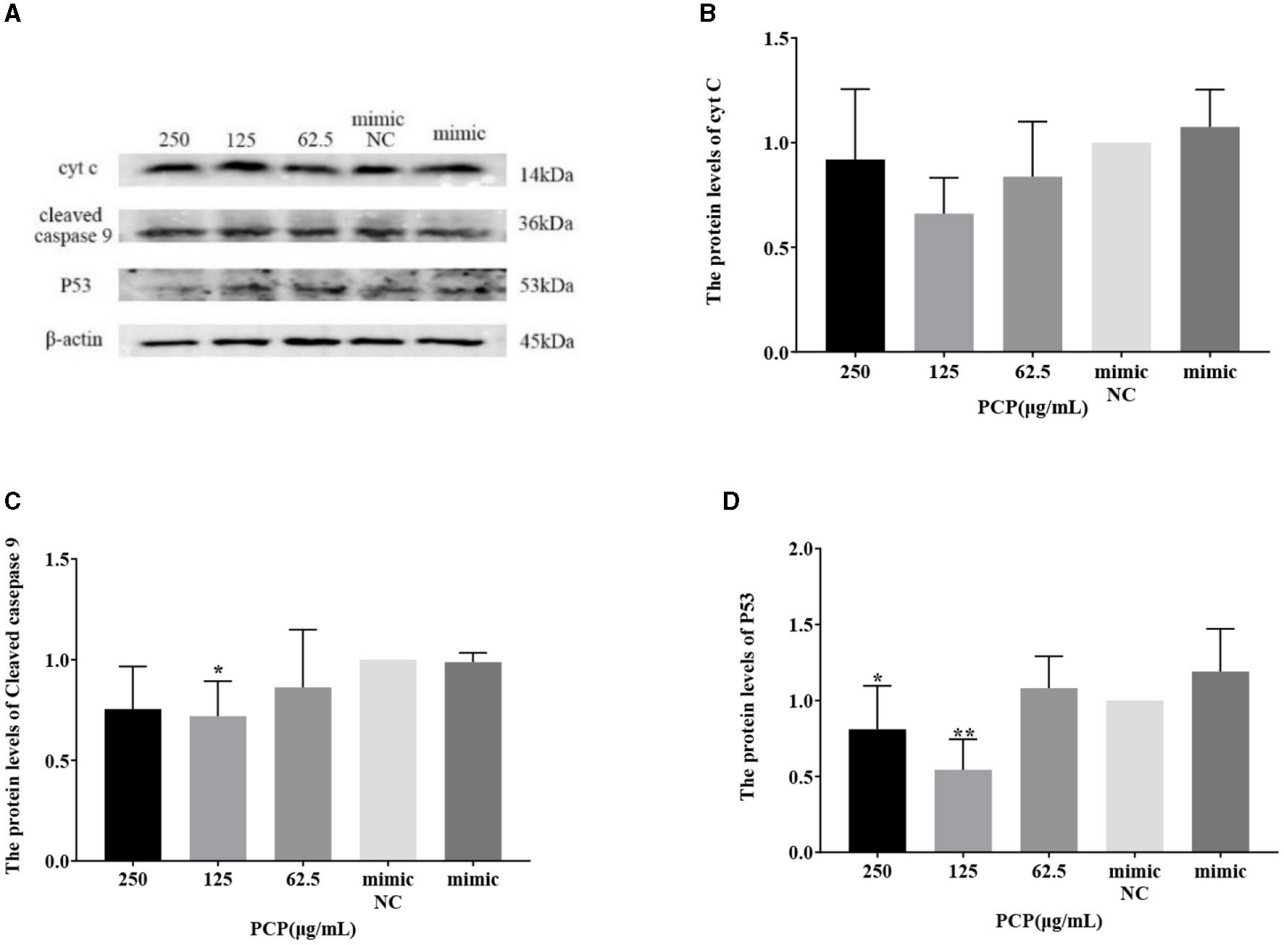
Effect of transfected miR-17 mimic on protein expression in cells. The protein expression levels of cyt C, caspase-9 and P53 were measured by Western blot. The PCP, mimic and mimic NC were treated with TGEV. **(A)** Protein bands; **(B)** The protein expression levels of cyt C (*n* = 3); **(C)** The protein expression levels of cleaved caspase-9 (*n* = 3); **(D)** The protein expression levels of P53 (*n* = 3). *P* < 0.05 was statistically significant to the mimic group, ^*^P < 0.05, ^**^P < 0.01. The data and analysis results were plotted as bar graphs using GraphPad Prism 7.00.

**Figure 10:**
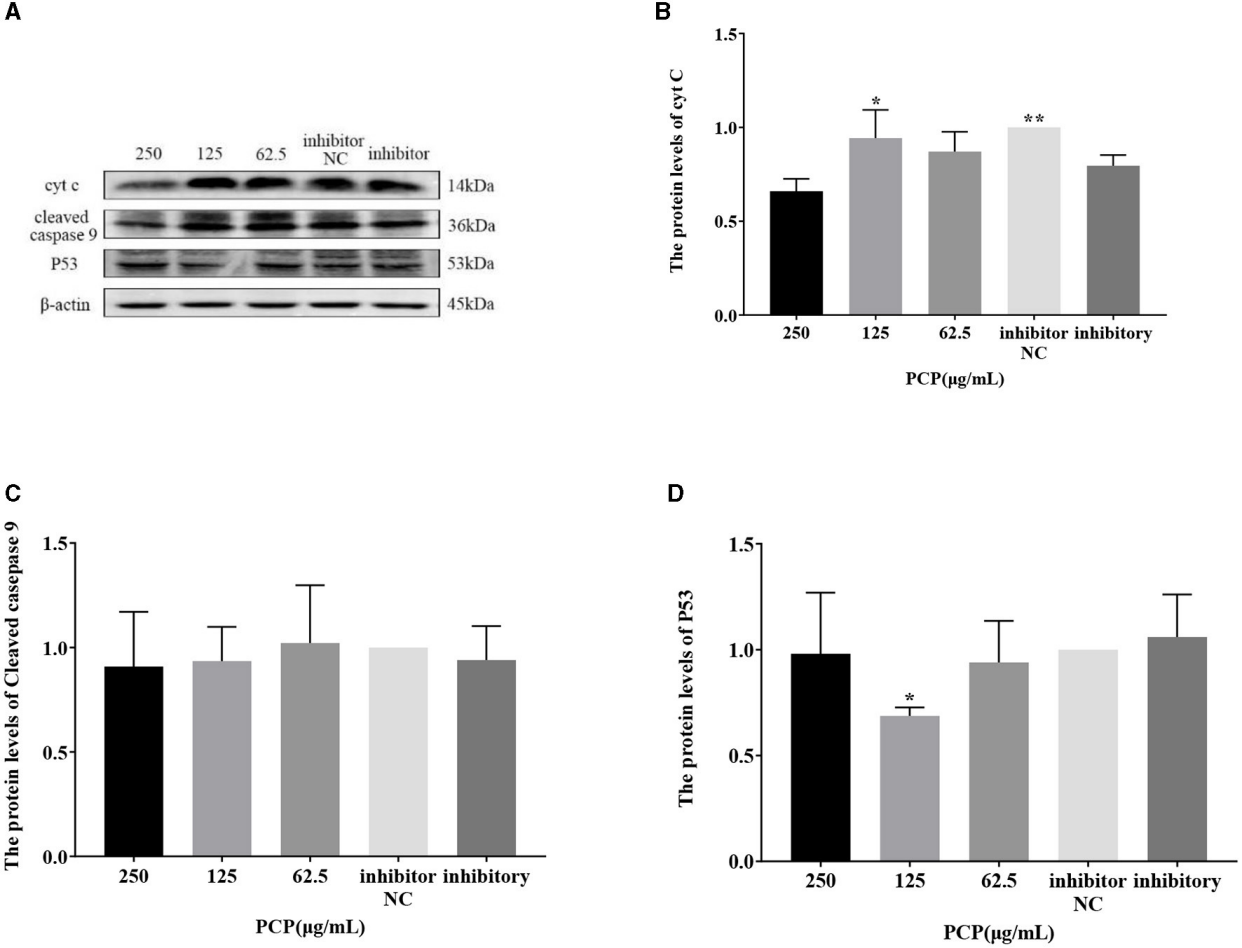
Effect of transfected miR-17 inhibitor on protein expression in cells. The protein expression levels of cyt C, caspase-9 and P53 were measured by Western blot. The PCP, inhibitory and inhibitor NC groups were treated with TGEV. **(A)** Protein bands; **(B)** The protein expression levels of cyt C (*n* = 3); **(C)** The protein expression levels of cleaved caspase-9 (*n* = 3); **(D)** The protein expression levels of P53 (*n* = 3). *P* < 0.05 was statistically significant to the inhibitory group, *P < 0.05, **P < 0.01. The data and analysis results were plotted as bar graphs using GraphPad Prism 7.00.

The results were shown in Figures 9, 10. Figures 9A, 10A show the protein bands of P53, cyt C and cleaved caspase9. After transfection of miR-17 mimic, the expression of cyt C (Figure 9B), cleaved caspase 9 (Figure 9C) and P53 (Figure 9D) in the mimic group was not significantly different from that in the mimic NC group. Compared with the mimic group, the expression of cyt C and cleaved caspase 9 was significantly inhibited in the PCP group of 125 μg/mL (Figures 9B, C) (*P* < 0.05). The expression of P53 was significantly inhibited in the PCP groups of 250 and 125 μg/mL (*P* < 0.001) (Figure 9C). After transfection of miR-17 inhibitor, the expression of cyt C in the inhibitor NC group was significantly higher than that in the inhibitory group (*P* < 0.01) (Figure 10B), while there was no significant difference in the expressions of cleaved caspase 9 (Figure 10C) and P53 (Figure 10D). Compared with the inhibitory group, the PCP group of 125 μg/mL significantly promoted expression of cyt C (Figure 10B) (*P* < 0.05); The expression of P53 was significantly inhibited in the PCP group of 125 μg/mL (Figure 10D) (*P* < 0.05). I the explanation indicated that the miR-17 inhibitor suppressed the expression of cyt C; At the same time, PCP restrained the expression of cyt C, cleaved caspase 9 and P53 in PK15 cells infected with TGEV following transfection of miR-17 mimic. Meanwhile, PCP restrained the expression of P53 and promoted the expression of cyt C after transfection of miR-17 inhibitor.

### 3.9 Effect of PCP on TGEV replication after miR-17 mimic or miR-17 inhibitor transfection

**Figure 11:**
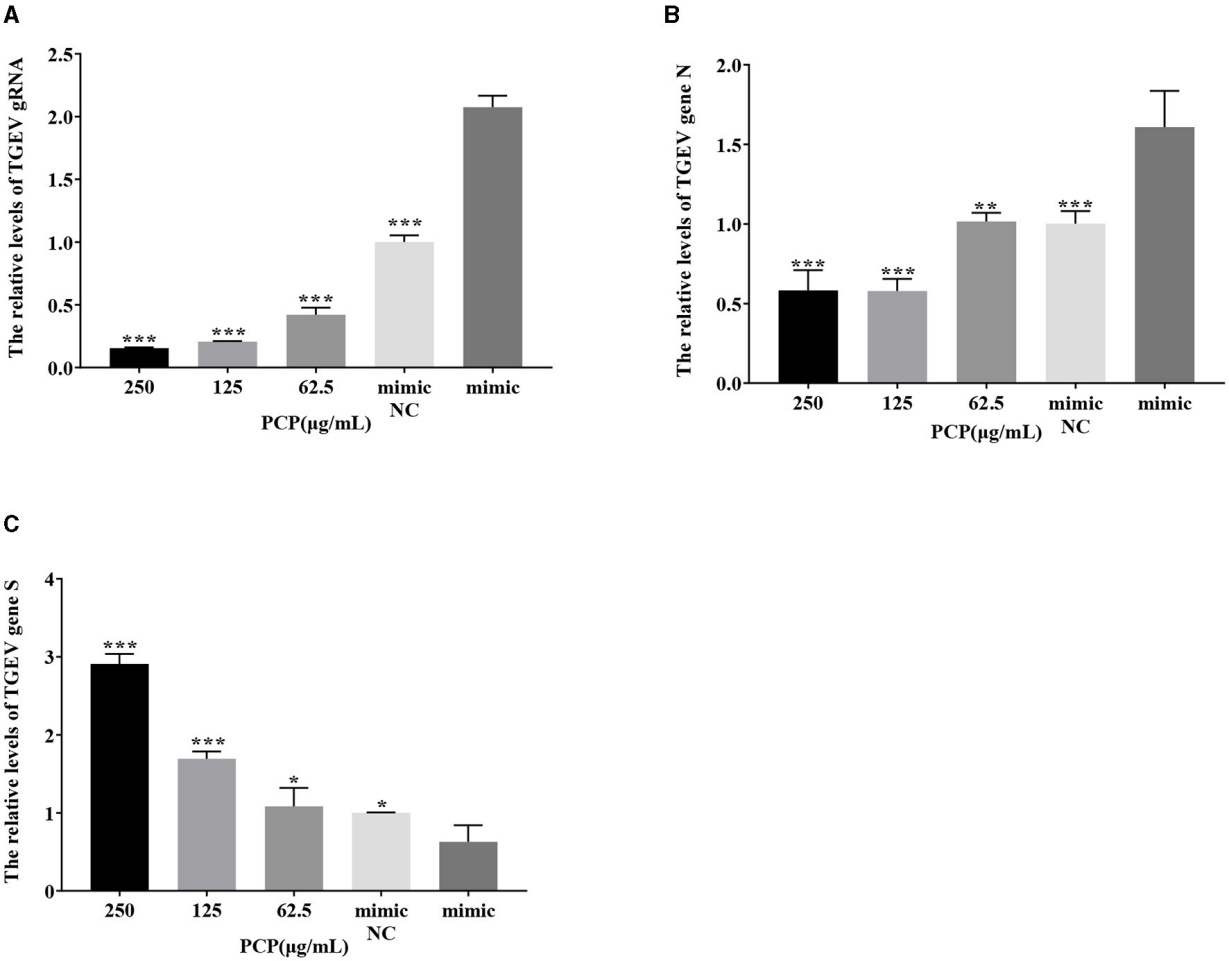
The effect of miR-17 mimic on TGEV replication. The mRNA expression levels of TGEV gRNA, gene N and gene S were measured by RT-qPCR. The PCP, mimic and mimic NC were treated with TGEV. **(A)** The mRNA expression levels of TGEV gRNA (*n* = 3); **(B)** The mRNA expression levels of TGEV gene N (*n* = 3); **(C)** The mRNA expression levels of TGEV gene S (*n* = 3). *P* < 0.05 was statistically significant to the mimic group, *P < 0.05, **P < 0.01, ***P < 0.001. The data and analysis results were plotted as bar graphs using GraphPad Prism 7.00.

**Figure 12:**
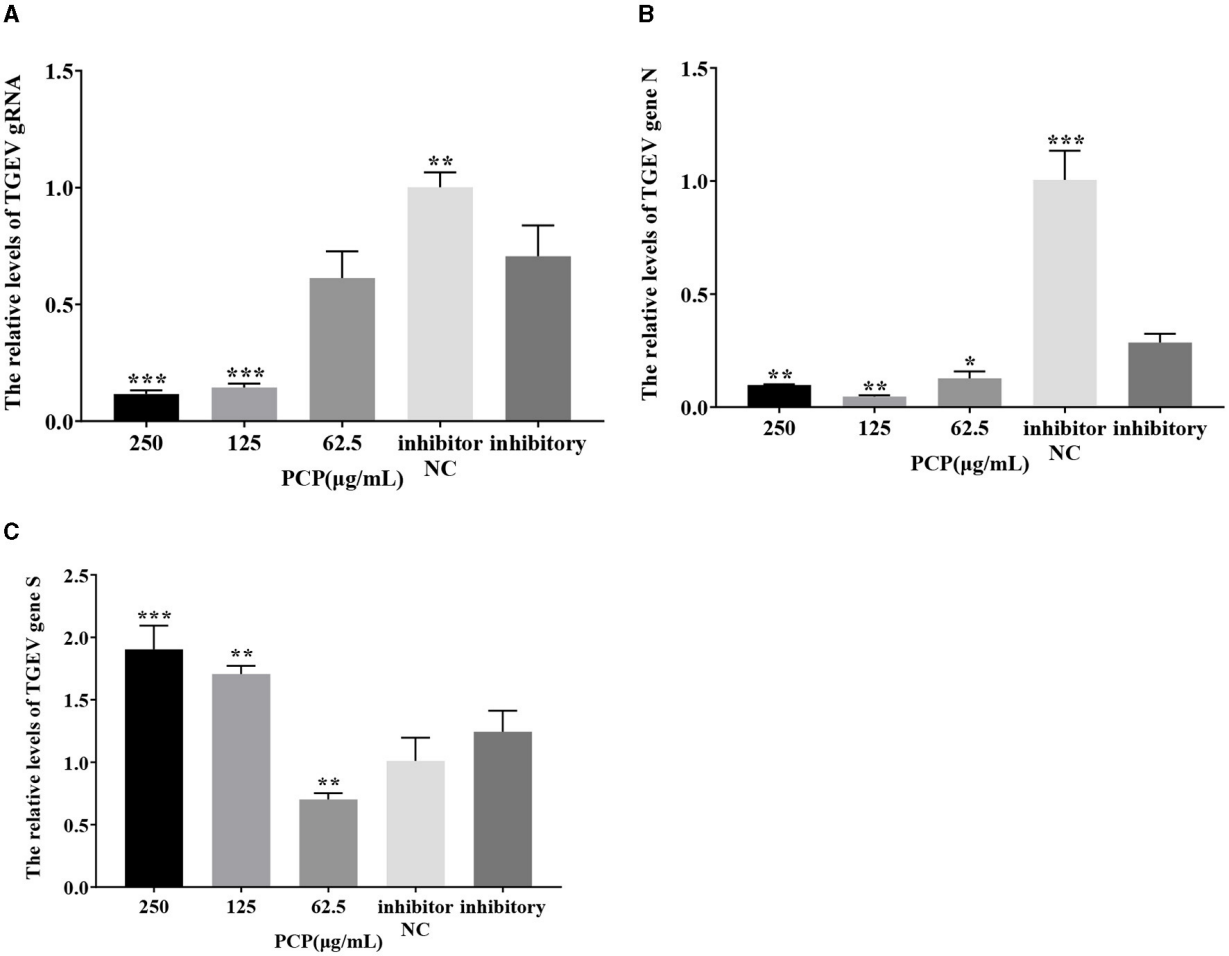
The effect of miR-17 inhibitor on TGEV replication. The mRNA expression levels of TGEV gRNA, gene N and gene S were measured by RT-qPCR. The PCP, inhibitory and inhibitor NC were treated with TGEV. **(A)** The mRNA expression levels of TGEV gRNA (*n* = 3); **(B)** The mRNA expression levels of TGEV gene N (*n* = 3); **(C)** The mRNA expression levels of TGEV gene S (*n* = 3). *P* < 0.05 was statistically significant to the inhibitory group, *P < 0.05, **P < 0.01, ***P < 0.001. The data and analysis results were plotted as bar graphs using GraphPad Prism 7.00.

The results were shown in Figures 11, 12. After transfection of miR-17 mimic, the relative expression of TGEV gRNA in the mimic NC group was significantly lower than that in the mimic group (*P* < 0.001) (Figure 11A), and the relative expression of TGEV gene N in the mimic NC group was significantly lower than that in the mimic group (*P* < 0.01) (Figure 11B). The relative expression of TGEV gene S was significantly higher than that in the mimic group (*P* < 0.05) (Figure 11C). Compared with the mimic group, the PCP groups of 250 μg/mL−62.5 μg/mL significantly inhibited the replication of TGEV gRNA (*P* < 0.001) (Figure 11A). The PCP groups at 250 and 125 μg/mL significantly inhibited TGEV gene N replication (*P* < 0.001) and TGEV gene N replication at 62.5 μg/mL (*P* < 0.01) (Figure 11B). PCP significantly promoted the replication of TGEV gene S at concentrations of 250 and 125 μg/mL (*P* < 0.001) and TGEV gene S replication at 62.5 μg/mL (*P* < 0.05) (Figure 11C). After transfection of miR-17 inhibitor, the relative expression of TGEV gRNA in the inhibitor NC group was significantly higher than that in the inhibitory group (*P* < 0.01) (Figure 12A). The relative expression of TGEV gene N in the inhibitor NC group was significantly higher than that in the inhibitory group (*P* < 0.01) (Figure 12B); There was no significant difference from TGEV gene S between inhibitor NC group and inhibitory group (Figure 12C). Compared with the inhibitory group, PCP significantly inhibited TGEV gRNA replication at concentrations of 250 and 125 μg/mL (*P* < 0.001) (Figure 12A). PCP significantly inhibited TGEV gene N replication at concentrations of 250 and 62.5 μg/mL (*P* < 0.05) and TGEV gene N replication at 125 μg/mL (*P* < 0.01) (Figure 12B). PCP significantly promoted the replication of TGEV gene S at the concentration of 250 μg/mL (*P* < 0.001) and 125 μg/mL (*P* < 0.01), and significantly inhibited the replication of TGEV gene S at the concentration of 62.5 μg/mL (*P* < 0.01) (Figure 12C). This indicated that miR-17 mimic promoted the replication of TGEV gRNA and gene N, while miR-17 inhibitor inhibited the replication of TGEV gRNA and gene N; At the same time, PCP inhibited the replication of TGEV gRNA and gene N after transfection of miR-17 mimic and inhibitor, promoting the replication of TGEV gene S after transfection with miR-17 mimic. After transfection with miR-17 inhibitor, the replication of TGEV gene S was promoted at high concentrations of PCP and inhibited at low concentrations.

## 4 Discussion

Virus infection poses a significant threat to both human and animal health. Consequently, the pursuit of safe, effective, and specific antiviral drugs remains a prominent area of research. Polysaccharides, as one of the main components of drugs, have multiple functions in living organisms. Studies have revealed that a multitude of polysaccharides have antiviral activity, while also possessing low toxicity and low resistance (19). According to lots of reports, *Isatidis Radix* polysaccharide have antiviral effects on pseudorabies virus (20), and *Glycyrrhiza* and *Panax otoginseng* polysaccharide could interfere with virus attachment and internalization, inhibit PRV adsorption and replication, and exert antiviral effects (21, 22). Polysaccharides present in algae could resist HIV-1, inhibit HSV-I and II, and reduce the infectivity of virus BoHV-1 strain Cooper and SuHV-1 strain Bartha (23). Therefore, polysaccharide, as an effective and low toxic antiviral component, has broad medicinal prospects. At present, drugs used to prevent or treat viral diseases are mainly Chinese herbal medicines, Chinese proprietary medicines and chemical drugs. Research has confirmed that some traditional Chinese medicines possess very strong antiviral effects. The single traditional Chinese medicines for clinical prevention and treatment of viral diseases, such as antipyretic drug *Isatidis radix, Scutellaria baicalensis, Coptis chinensis, Forsythia suspensa, Flos lonicerae*, etc. (24–28). The main proprietary Chinese medicines include *Tongxuan Lifei Pills, Jiuwei Qianghuo Decoction, Yinqiao Jiedu Pills, Ganke Shuangqing Capsule, Shuanghuanglian Injection*, etc. (29–33). Therefore, enriching the types and mechanisms of antiviral drugs could provide more safety guarantees for viral infections. The *Polygonum Cillinerve* Polysaccharide has been proven to have antiviral activity through this study, and further research is needed on their mechanism of action against TGEV.

Animal viral infectious diseases have been demonstrated to be associated with apoptosis (34–36), in the case of TGEV infection, cells underwent apoptosis through both endogenous and exogenous pathways to inhibit virus replication. The endogenous pathway of apoptosis is initiated by mitochondria, and viral infection can cause mitochondrial damage (37, 38). The main role of mitochondria is to rely on OXPHOS to produce ATP for cells, and the byproduct of this process is the production of ROS (39). Homeostasis of ROS is essential for maintaining normal biological processes. The role of ROS in virus-induced apoptosis has been demonstrated by numerous studies. For example, after parvovirus H-1 infects the body, apoptosis was induced by mediating ROS accumulation (40), and levistilide A can inhibit the replication of PEDV by inducing the production of ROS (41). At the same time, there are also studies which have proved that miRNA could affect the production of ROS, such as overexpression of miR-27 could inhibit the production of ROS (42). Therefore, after transfection of miRNA, ROS was detected to explore the effects of PCP and TGEV. The results showed that PCP inhibited the production of ROS induced by TGEV after transfection of miR-17.

The mitochondrial membrane potential (ΔΨm) generated by the proton pump (complexes I, III, and IV) constitutes a crucial element in the energy storage process during oxidative phosphorylation (43). ΔΨm levels in cells remain relatively stable, reflecting normal physiological activity (44–46). However, persistent alterations in these two factors can have detrimental effects, and a prolonged decrease or increase in ΔΨm compared to normal levels may result in an unnecessary decline in cell viability and serve as the underlying cause of various pathologies (47). In this study, after transfection of miRNA, each group of cells was loaded with JC-1 fluorescent probes, and the decrease in cell membrane potential could be easily detected by the transition of JC-1 from red fluorescence to green fluorescence, which could also be used as an early detection index of apoptosis. Statistical analysis of the relative ratio of red and green fluorescence revealed that JC-1 staining could demonstrate that PCP could protect PK15 cells infected with TGEV from a decrease in mitochondrial membrane potential after miR-17 transfection, and could significantly increase the mitochondrial membrane potential at high concentrations (*P* < 0.05).

Hoechst 33258 is a blue fluorescent dye that can penetrate cell membranes, embed into double stranded DNA and release intense blue fluorescence. It was low cytotoxic and widely used to assess cell cycle and apoptosis (48). During apoptosis, chromatin condenses white under fluorescence microscopy. Studies have proved that TCM formulas or active ingredients in TCM could regulate apoptosis by modulating miRNA. For example, Ditan Huoxue Shubi Decoction can upregulate the expression of miR-148, down-regulate the expression of TXNIP, and reduce the level of H/R induced autophagy and apoptosis of cardiomyocytes (49). *Platycodon* polysaccharides ameliorate respiratory syncytial virus-induced apoptosis and inflammation responses in epithelial cells by activating miR-181 mediated Hippo and SIRT1 pathways (50). This was similar to the results of this study, where nuclear bleaching in the PCP group after transfection of miR-17 was dose-dependent with drug concentration, suggesting that high concentrations of the drug inhibited apoptosis. Furthermore, the results of the experiment also showed that PCP could inhibit TGEV-induced cell apoptosis after transfection of miR-17 alone. Thus, it was shown that PCP was able to inhibit TGEV-induced apoptosis by down-regulating the expression of miR-17.

The study found that miR-17 was highly expressed in patients with multiple myeloma and colon cancer, and low expressed in skin lesions and peripheral blood of patients with gastric stromal tumors and psoriasis vulgaris, and was involved in regulating the development of porcine ovaries from follicular stage to luteal stage, adipocyte differentiation and porcine adipose deposition (51). It has not been shown to be involved in the regulation of viral infection. In this study, after high-throughput sequencing, it was found that miR-17 was differentially expressed during apoptosis caused by PCP and TGEV infection, so after transfection of miR-17 mimic and inhibitor, the mRNA expression of caspase 9 in the mimic group was lower than that in the NC group, but there was no significant difference (Figure 7). The mRNA expression of caspase 9 in the inhibitor group was significantly higher than that in the NC group (*P* < 0.01) (Figure 8), indicating that under ideal circumstances, inhibitor binds to miR-17 and cannot bind to the 3' UTR of caspase 9, or binds less, resulting in high mRNA expression of caspase 9. Therefore, it is speculated that miR-17 might be the target gene for caspase 9. After treated with PCP, there were significant differences in mRNA expression levels of the three nodes on the apoptosis pathway (P53, cyt C, caspase 9) (Figures 7, 8), indicating that PCP promoted the mRNA expression of apoptosis-related factors. But at the protein level, it showed different results. MiR-17 had no effect on the protein level of caspase 9, possibly due to various regulatory mechanisms from genes to protein function, including but not limited to DNA modification, histone modification, transcription element regulation, RNA modification, RNA editing, protein post-translational modification and so on. After treated with PCP and transfected of the inhibitor, the protein level of P53 decreased, while the protein level of cyt C increased. However, caspase 9, as a downstream effector molecule, did not show any difference; At the same time, PCP significantly inhibited the protein levels of P53, cyt C, and cleaved caspase 9 after transfection with mimic (Figures 9, 10). This experiment suggests that caspase 9 might be a target gene for miR-17, and further validation was needed to confirm this relationship. However, PCP after transfection of miR-17 might inhibit apoptosis caused by TGEV via the P53 pathway.

Since cellular miRNAs are involved in the life cycle of many viruses, their importance in virus-host interactions is becoming increasingly evident in addition to their key role in regulating gene expression in normal cells (52–54). At the same time, studies have confirmed that TGEV infection initiates the mechanism of apoptosis, and further research is still needed on how apoptosis affects TGEV proliferation and spread. Therefore, in this study, the effects of differentially expressed miR-17 on viral genome and subgenomic replication during the process of PCP anti-TGEV were investigated, and the mechanism of miRNA in PCP anti-TGEV process was further explored. It was found that overexpression of miR-17 facilitated TGEV replication, which was similar to the study of Castillo et al. (3) overexpression of miRNA-133 could influenced the replication of DENV-2, affect the percentage of infected cells and the number of viral RNA copies (3, 55). TGEV is an important pathogen for pigs that can cause severe diarrhea in pigs (especially newborn piglets) and huge economic losses. In addition, the results of this study also demonstrate that host miRNAs could affect the replication of TGEV gene N, gene S, and gRNA, and PCP might inhibit TGEV replication by down-regulating miR-17 expression. These findings further highlight the important role of mammalian miRNA in antiviral response and further enrich the antiviral mechanism of traditional Chinese medicine, which may be of great significance for the molecular mechanism of miRNA in the anti-TGEV process of traditional Chinese medicine.

## 5 Conclusion

This study demonstrated that PCP inhibited TGEV induced cell morphological changes, ROS production, the reduction of mitochondrial membrane potential, and cell apoptosis after transfection with miR-17. After transfection of miR-17, PCP could inhibit the apoptosis of PK15 cells induced by TGEV through P53 pathway. In addition, miR-17 affected TGEV replication in PK15 cells, and PCP might inhibit TGEV replication by down-regulating miR-17 expression.

## Data availability statement

The original contributions presented in the study are included in the article/Supplementary material, further inquiries can be directed to the corresponding authors.

## Ethics statement

The animal study was approved by Animal Ethical and Welfare Committee, Northwest A&F University, China. The study was conducted in accordance with the local legislation and institutional requirements.

## Author contributions

XD: Data curation, Formal analysis, Investigation, Methodology, Visualization, Writing – original draft. MX: Writing – original draft, Writing – review & editing. YW: Writing – original draft. NL: Validation, Writing – review & editing. XW: Validation, Writing – review & editing. YL: Writing – review & editing. WZ: Writing – review & editing. WM: Writing – review & editing. LM: Supervision, Writing – review & editing. YF: Conceptualization, Funding acquisition, Project administration, Supervision, Writing – review & editing.
