# Supplementary material for: Effect of miR-17 on Polygonum Cillinerve polysaccharide against transmissible gastroenteritis virus
Source: Front Vet Sci. 2024 Feb 20;11:1360102. doi: 10.3389/fvets.2024.1360102 (PMC10912159; doi:10.3389/fvets.2024.1360102)
Supplement: Supplementary file 1 [file Data_Sheet_1.docx]

# **Supplement**

## 1 Methods

### 1.1 Cell viability

PK15 cells were laid on the 12 well cell culture plate and changed to basic culture medium for starving for 30 min when the cells grew to 30%-40%. Following the process in the miRNA product manual and Lipofectamine 2000 transfection reagent manual, miR-17 mimic and its corresponding NC were transfected separately at the concentration of 50 nM; The miR-17 inhibitor and its corresponding NC were transfected separately at the concentration of 100 nM. After 4 h of transfection, the cells were continued to culture with complete culture medium for 24 h, then TGEV and different concentrations of PCP (62.5, 125, 250 μg/mL) were added into the cells transfected with mimic or inhibitor. The mimic NC, inhibitor NC, mimic control and inhibitor control were added with the same volumes of TGEV. Mimic blank and inhibitor blank groups were added with culture medium. The cells were treated for 1.5 h, then washed with PBS three times, and the maintenance medium was replaced to continue cultivation. After 44 h, the culture medium was collected and the cells were added 100 μL fresh complete culture medium and 30 μL MTT at the concentration of 500 μg/mL. After 4 h, the culture medium was removed and the cells were added with 150 μL DMSO to each well and the absorbance was detected at 570 nm.

## 2 Results

### 2.1 Effect of TGEV on cell viability in PK15 cells transfected with miR-17 mimic or inhibitor

Figure S1 showed the activity of PK15 cells transfected with miR-17 mimic, inhibitor, and their NC after treatment with PCP and TGEV. The results showed that TGEV significantly inhibited the activity of PK15 cells (P < 0.001), and after transfection with miR-17 mimic or inhibitor, PCP inhibited the damaging effect of TGEV on PK15 cells in the concentration dependent manner (Figure 1SA and B) (P < 0.001).

**Figure 1S. The activity of PK15 cells.** The activity of PK15 cells were detected by MTT. The PCP groups were first transfected with miR-17 mimic or inhibitor, and then treated with TGEV and PCP for 1.5 h; Mimic, mimic NC, inhibitory, and inhibitor NC were only transfected with corresponding miRNAs and treated with TGEV for 1.5 h, while the blank group was not treated. A: cell viability of PCP groups (250, 125, 62.5 μg/mL) transfected with miR-17 mimic, mimic group, mimic NC group and mimic blank group; B: cell viability of PCP groups (250, 125, 62.5 μg/mL) transfected with miR-17 inhibitor, inhibitory group, inhibitor NC group and inhibitor blank group. P < 0.05 was statistically significant, P* < 0.05, P** < 0.01, P*** < 0.001. The data and analysis results were plotted as bar graphs using GraphPad Prism 7.00.
